# Analysis for the Influence of ABR Sensitivity on PTT-Based Cuff-Less Blood Pressure Estimation before and after Exercise

**DOI:** 10.1155/2018/5396030

**Published:** 2018-10-08

**Authors:** Yang Xu, Peng Ping, Dong Wang, Weigong Zhang

**Affiliations:** School of Instrument Science and Engineering at Southeast University, Nanjing, China

## Abstract

An accurate and continuous measurement of blood pressure (BP) is of great importance for the prognosis of some cardiovascular diseases in out-of-hospital settings. Pulse transit time (PTT) is a well-known cardiovascular parameter which is highly correlated with BP and has been widely applied in the estimation of continuous BP. However, due to the complexity of cardiovascular system, the accuracy of PTT-based BP estimation is still unsatisfactory. Recent studies indicate that, for the subjects before and after exercise, PTT can track the high-frequency BP oscillation (HF-BP) well, but is inadequate to follow the low-frequency BP variance (LF-BP). Unfortunately, the cause for this failure of PTT in LF-BP estimation is still unclear. Based on these previous researches, we investigated the cause behind this failure of PTT in LF-BP estimation. The heart rate- (HR-) related arterial baroreflex (ABR) model was introduced to analyze the failure of PTT in LF-BP estimation. Data from 42 healthy volunteers before and after exercise were collected to evaluate the correlation between the ABR sensitivity and the estimation error of PTT-based BP in LF and HF components. In the correlation plot, an obvious difference was observed between the LF and HF groups. The correlation coefficient *r* for the ABR sensitivity with the estimation error of systolic BP (SBP) and diastolic BP (DBP) in LF was 0.817 ± 0.038 and 0.757 ± 0.069, respectively. However, those correlation coefficient *r* for the ABR sensitivity with the estimation error of SBP and DBP in HF was only 0.403 ± 0.145 and 0.274 ± 0.154, respectively. These results indicated that there is an ABR-related complex LF autonomic regulation mechanism on BP, PTT, and HR, which influences the effect of PTT in LF-BP estimation.

## 1. Introduction

Blood pressure (BP) is a vital sign which is defined as the pressure of circulating blood on the walls of blood vessels. In morphology, BP is usually expressed in terms of the systolic BP (maximum during one heartbeat) over diastolic BP (minimum in between two heartbeats). Continuous blood pressure monitoring is of great clinical significance which could provide a long-term character of the cardiac system. It is important to the diagnosis of cardiac diseases. However, the traditional BP monitoring involves the inflating and deflating of a cuff which causes unavoidable intermittent monitoring of the blood pressure. Because of this shortcoming of cuff-based BP monitoring and the profound need for continuous blood pressure monitoring, the scientific community has paid a lot of attention to the cuff-less continuous BP estimation methods in recent decades [1].

Among these cuff-less BP measurement methods, the PTT*-*based method has been extensively investigated in clinical settings. A mountain of research studies has demonstrated that there is a high correlation between BP and PTT [2–5]. PTT is a cardiovascular parameter which is defined as the time delay for the pressure wave to travel from a proximal point to a distal point in the arterial within the same cardiac cycle. It could be easily calculated from the feature points of ECG and PPG. The principal fundamental behind this method is that the blood flow in the arteries is physically modeled as the propagation of pressure waves inside elastic tubes. In this way, the physical characteristics of blood flow wave in arteries could be described by the Moens–Korteweg (MK) equation as follows [6]:

(1) $$\begin{array}{c} \text{PWV}=\frac{L}{\text{PTT}}=\sqrt{\frac{Gh}{\rho 2r}}, \end{array}$$

where *L*, *G*, *h*, *r*, and *ρ* are the length of the vessel, the elastic modulus of the tube wall, the vessel wall thickness, the vessel radius, and the constant blood density, respectively. PWV is the velocity of the blood flow wave propagating in the entire arterial tree, which is inversely related with PTT. *G* is exponentially correlated with BP through

(2) $$\begin{array}{c} G=G_{0}e^{\gamma p}, \end{array}$$

where *G*_0_ is the elastic modulus at zero pressure, *γ* is a coefficient depending on a particular vessel, and *P* is BP. The MK equation and its variants could also be simplified into different mathematical models, in which the relevant hemodynamic parameters (blood density, vessel radius, etc.,) were converted into a number of individual-specific model parameters (see Section 2.1 for details). Therefore, with an initial calibration of these individual-specific model parameters, the PTT-BP relationship is obtained. Then, measuring PTT could offer a continuous and cuff-less BP monitoring [2].

Over the past 20 years, various calibration models of the BP-PTT relationship have been proposed to achieve the continuous BP measurement with PTT [7–21]. Chen et al. [7] established a mathematical model involving the patients' ages and genders. It indicated that this model could capture the PTT-DBP relationship for subjects on a wider range. Gesche et al. [11] developed a one-point calibration model for the PTT-BP relationship, which needs only one measurement of BP using a cuff-based reference. The results indicated that the SBP calculated from PTT correlates significantly with the cuff-based SBP (*r*=0.83). Esmaili et al. [18] proposed a PTT-BP nonlinear model for the accurate estimation of both SBP and DBP. It attained a high accuracy with evaluated error and variance of 0.12 ± 6.15 mmHg for SBP and 1.31 ± 5.36 mmHg for DBP.

Although the PTT-based method has been considered as the most promising cuff-less continuous BP monitoring technique, there are still several problems that need to be solved before its widespread application [8]. The major and most important challenge is that the accuracy of PTT-based BP estimation is still not satisfactory enough. The possible influence factors include: arterial compliance, cardiac output, peripheral resistance, and blood volume [10]. However, due to the complexity of the cardiovascular system, the influences of these physiological factors on the PTT-based BP are hard to be evaluated. It is impracticable to employ these factors directly to improve the accuracy of PTT-based BP estimation. The possible solution is, based on the experiment phenomenon, to introduce the applicable regulation models or parameters into the PTT-BP calibration model to weaken the influences from other physiological factors.

From the differences of influence factors, there are two special frequency bands of the BP changes [22–24]: (1) the high-frequency (HF, 0.2–0.35 Hz) BP oscillation influenced by the physical factors (e.g., respiration) and (2) the low-frequency (LF, 0.1–0.15 Hz) BP variance under the control of the autonomic nervous system (ANS). Recently, various researches [25–27] have investigated the time-frequency correlation between the variability of PTT, HR, and BP before and after exercise. The results indicate that, relatively speaking, PTT can track the HF BP oscillation well, but inadequate to follow the LF BP variance (see Section 2.3 for details). From the experimental results, this poor performance of PTT in LF-BP estimation is considered as a reason for the nonaccuracy of PTT-based BP estimation [10]. However, the cause for this failure of PTT in LF-BP estimation is still unknown.

Based on these previous researches, we try to introduce the ABR regulation model [28] (arterial baroreflex, a major BP-related autonomic nervous regulation mechanism, see section 2.2 for details) to analyze this failure of PTT in LF-BP estimation in this research. From the view of the ABR model and the interaction between the cardiovascular parameters, it was found that the ABR sensitivity may influence the estimation accuracy of PTT-based LF-BP. Then, synchronous physiological data (BP, PPG, and ECG) from 42 subjects before and after exercises were collected to quantitatively analyze the influence of ABR sensitivity on the PTT-based HF- and LF-BP with the help of VMD (variation mode decomposition, a time-frequency analysis technique). The correlation between the ABR sensitivity and the estimation error of PTT-based HF- and LF-BP was evaluated. The aim was to analyze the cause behind failure of PTT in LF-BP estimation in the experimental condition. It is expected that the results of this work may help to construct a better PTT-BP calibration model in practice.

The rest of this paper is organized as follows.  Section 2 introduces the backgrounds of this paper including the current PTT-based calibration model, the ABR model, the time-frequency correlation between BP, HR, and PTT before and after exercise, and the qualitative analysis of the possible cause for the failure of PTT in LF-BP estimation.  Section 3 explains the quantitative analysis methodology including the data collection and preprocessing, the calculation method of ABR sensitivity, and the correlation analyses between the ABR sensitivity and the PTT-based BP estimation error.  Section 4 presents and discusses the results of the experiments. The conclusion and further works are given in Section 5.

## 2. Backgrounds

### 2.1. The Calibration Models of PTT-BP Relationship

In the PTT-based BP estimation method, the arterial vessels are physically modeled as an elastic tube. The pressure wave propagating on the vessels follows a function of the pulse propagating position and time (*x* and *t*, respectively) as [4]

(3) $$\begin{array}{c} P\left(x,t\right)=f\left(x\pm \frac{t}{\sqrt{LC\left(P\right)}}\right), \end{array}$$

where *L* is a constant that represents the arterial inertance per unit length. The vessel compliance *C* is defined as the rate of tube cross section changes in terms of blood pressure *P* as follows:

(4) $$\begin{array}{c} C\left(P\right)=\frac{A_{m}}{\pi P_{1}\left[1+{\left(\left(P-P_{0}\right)/P_{1}\right)}^{2}\right]}, \end{array}$$

where *P*_0_, *P*_1_, and *A*_*m*_ are subject-specific parameters [5]. Accordingly, the PWV is equal to ${\left(\sqrt{LC\left(P\right)}\right)}^{-1}$, and PTT is the time interval for the pressure wave traversing a tube of length *l*, which is expressed as:

(5) $$\begin{array}{c} \text{PTT}=l\sqrt{LC\left(P\right)}. \end{array}$$

Then, combining equations (3)–(5) with the aforementioned equations (1) and (2), the two most popular calibration models in the literature could be obtained. By substituting (1) into (2), a popular calibration model is given as follows [7]:

(6) $$\begin{array}{c} \text{BP}=K_{1}\ln\left(\text{PTT}\right)+K_{2}. \end{array}$$

The other popular physical calibration model is derived by substituting (4) into (5), given as follows [8]:

(7) $$\begin{array}{c} \text{BP}=\frac{K_{1}}{\text{PTT}+K_{2}}, \end{array}$$

where *K*_1_and *K*_2_ are unknown subject-specific parameters which are obtained by fitting reference BP with PTT in the regression model.

Experimental studies have shown that 1/PTT, rather than PTT, is linearly related to BP [11, 12]. In other words, model (7) performs better in PTT-BP fitting than model (6). However, no matter model (6) or model (7) are the MK equation-based models, which could only indicate the rhythmic HF-BP oscillation that was caused by the physical activity [6]. The BP changes are not only HF-oscillating caused by the exogenous driving but also LF-fluctuating due to the autonomic nervous regulation [23].

### 2.2. Arterial Baroreflex Regulation Model

**Figure 1:**
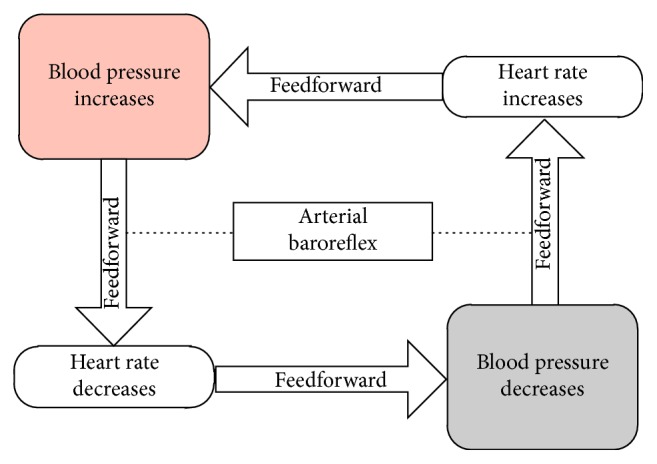
The simplified diagram of ABR.

Arterial baroreflex (ABR) is a major autonomic nervous regulation mechanism which is responsible to stabilize BP [28]. The baroreceptors, locating along all major arteries of the human body, sense the BP changes and deliver a signal to the autonomous nervous system (ANS). Then, the ANS stabilizes BP through a feedback regulation mechanism by HR like this: decrease (increase) in BP leads to the consequent reduction (enhancement) in the HR. Meanwhile, the increase (decrease) in HR causes the increase (decrease) in BP as a direct feedforward effect. Therefore, the ABR mechanism could be described by a simplified double-loop feedback diagram (Figure 1).

**Figure 2:**
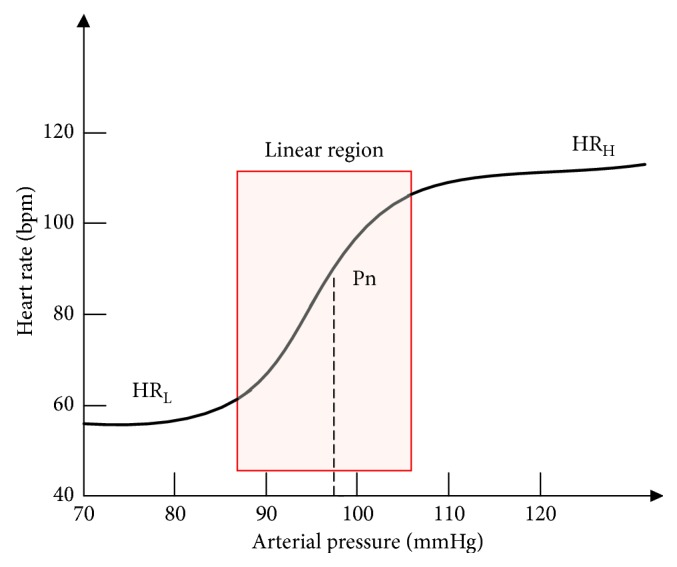
The schematic diagram of the overall ABR regulation model.

The overall ABR regulation nonlinearly drives the heart rate on the basis of the arterial pressure, which follows a logistic model [28]:

(8) $$\begin{array}{c} \text{HR}\left(P\right)={\text{HR}}_{l}+\frac{{\text{HR}}_{h}-{\text{HR}}_{l}}{1+e^{-\varepsilon \left(P-P_{n}\right)}}, \end{array}$$

where, HR_*l*_ and HR_*h*_ are the lower and upper levels of heart rate, respectively, *P*_*n*_ denotes the arterial pressure at the midpoint of the heart rate range, and *ε* determines the slope of the linear region (or sensitive region) in the overall ABR regulation curve (Figure 2).

### 2.3. The Time-Frequency Correlation Between BP, HR, and PTT before and after Exercise

In the past studies, numerous studies [25–27] have investigated the time-frequency correlation between the variability of PTT, HR, and BP before and after exercise. The variability of these physiological data is defined as the variation between two consecutive data points. Drinnan et al. [25] analyzed the cross-correlation function between PTT and HR from 15 normal healthy subjects during paced respiration. The results suggest that the high HRV would significantly influence the PTTV. This relationship between HRV and PTTV is negatively correlated, i.e.,

(9) $$\begin{array}{c} \text{HRV}↑\gg \text{PTTV}↓. \end{array}$$

Recently, using the recursive autoregressive model, Ma and Zhang [26] and Liu et al. [27] investigated the time-frequency correlation between BP, HR, and PTT in the LF and HF components for the subjects before and after exercise. The results reveal that PTT is highly correlated with HF-BP changes, but insignificantly correlated with LF-BP changes. However, the cause behind this phenomenon is still unclear. The possible reason is the mediation mechanisms of ANS on the cardiovascular system and the inherent correlations between the cardiovascular parameters.

### 2.4. Qualitative Analysis for the Failure of PTT in LF-BP Estimation

Based on these research studies and the ABR model, a qualitative analysis for the ineffective of PTT in LF-BP estimation before and after exercise was proposed here.

As shown in Figure 2, under the sensitive ABR regulation (the red box region), HR is increasing rapidly (high HRV) while BP is slow varying (LF-BP changes). As aforementioned in (9), PTTV is highly coupled with HRV. In this scenario, the high HRV significantly influences the fluctuation of PTT. The PTT would change unpredictably that may no longer follow the PTT-BP relationship model in (6) or (7), and leads to the failure of PTT in the estimation of LF-BP changes. On the other hand, under the nonsensitive ABR (region outside the red box), HR is slow varying (low HRV) as BP is increasing rapidly (HF-BP). Therefore, PTT is less affected by the HRV during the nonsensitive ABR regulation and predicts the HF-BP well. In brief, the high HRV may have unexpected effects on PTT under the ABR regulation. These ABR-model-based qualitative analysis results could explain the poor performance of PTT in LF-BP estimation well. It inspired us that ABR regulation is possibly the main cause for the failure of PTT in LF-BP estimation before and after exercise. However, further quantitative analysis should be implemented to confirm that. In the following sections, we investigated the correlation between the ABR sensitivity and the estimation error of PTT-based BP in HF-band and LF-band separately.

## 3. Methodology

### 3.1. Data Collection and Protocol

In our previous research [29], synchronous ECG, PPG, and reference BP data were collected on 42 healthy adults (21 males) with a mean age of 25.6 ± 2.1 years (range 21–31 years) from 8:00 to 11:00 a.m. in a quiet environment and at a constant room temperature of 22–25°C. These volunteers were nonsmokers with no history of cardiovascular diseases (CVDs) and no caffeine ingestion 6 h prior to the examination. Reference BP including systolic blood pressure (SBP) and diastolic blood pressure (DBP) was measured by Finapres (Finapres Medical System), a noninvasive BP measurement system, with the finger cuff on the right thumb and brachial cuff on the right upper arm. ECG and PPG were acquired with lead-II ECG electrode and PPG sensor (Biopac Systems) on left middle finger, respectively. All the data collection was performed with subjects in sitting position before and after a treadmill running exercise. Treadmill running exercise is a commonly used drugless method to increase the subject's BP variation range in trials [30]. The signals were recorded at the sampling rate of 1000 Hz. The ECG signal was filtered with a 0.5 Hz Butterworth high-pass filter and a 35 Hz Butterworth low-pass filter. The PPG signal was filtered with a 0.05 Hz Butterworth high-pass filter and a 10 Hz Butterworth low-pass filter. These filter parameters are the default setting that is recommended by the Biopac physiological data acquisition system.  Table 1 summarized the whole experimental procedure for each volunteer. In total, 3 ∗ 42 ∗ 20-min ECG, PPG, and reference BP signal were collected.

### 3.2. Parameter Extraction and Data Processing

**Figure 3:**
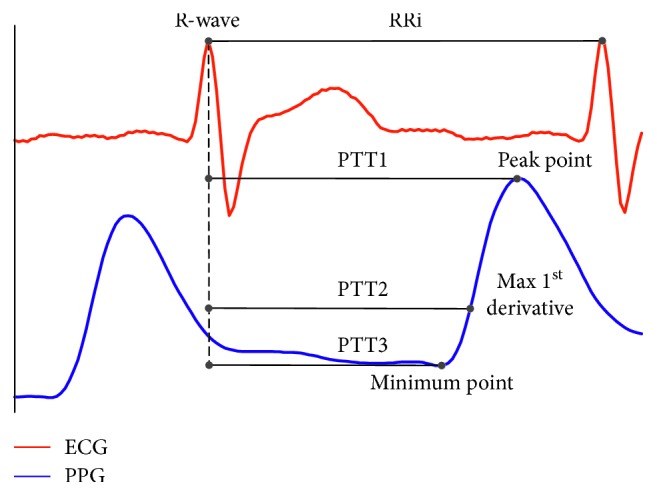
The definition of PTT and RR interval.

PTT is usually calculated as the time interval between the ECG R-waveform and (1) the peak point of PPG (PTT1), (2) the maximal first derivate point of PPG (PTT2), or (3) the minimum point of PPG (PTT3) among RR intervals (RRi) in the same cardiac cycle (see Figure 3). However, due to the motion artifact, the peak point or the minimum point of PPG is easy to deform, which influences the measurement of PTT1 and PTT3 in the experiment. Therefore, we chose the relatively more robust PTT2 [6] as the measurement of PTT to ensure the test results, here. Then, the obtained PTT was applied to match the reference SBP and DBP with the calibration model (7), which is recommended by Mukkamala et al. [6]. The PTT-BP relationship model was been adaptively trained with the ordinary least squares (OLS) algorithm which can be mathematically expressed as an optimization problem as follows:

(10) $$\begin{array}{c} \min_{\omega }{\left\|X\omega^{T}-BP_{\text{ref}}\right\|}_{2}^{2}, \end{array}$$

where *X*=(1/*PTT*, 0), *ω*=(*K*_1_, *K*_2_) as given in (7) and BP_ref_ is the referenced SBP and DBP.

In order to analyze the estimation accuracy of PTT-based BP in LF and HF range separately, VMD technology was utilized to decompose the estimated BP and reference BP (including SBP and DBP) signal. VMD is a newly developed time-frequency analysis technique proposed by Dragomiretskiy and Zosso [31]. Using VMD, the signal could be adaptively decomposed into an ensemble of *N* (*N* > 1) band-limited modes (BLMs) without the need of setting frequency range artificially. Each BLM compacts around a center frequency *ω*_*k*_ determined by the signal itself. The bandwidth for each BLM is calculated with the L2-norm of the gradient of its Hilbert transformed signal. Thus, these BLM could capture the intrinsic characters of the signal in the time-frequency domain without artificial influence. To benefit from this unique property of BLM, VMD technology has been widely used in the biosignal processing [32–34]. Here, the reference and estimated BP signals were decomposed into LF and HF modes. This process was implemented with the help of VMD Matlab toolbox software programmed by Dragomiretskiy and Zosso [31].

**Figure 4:**
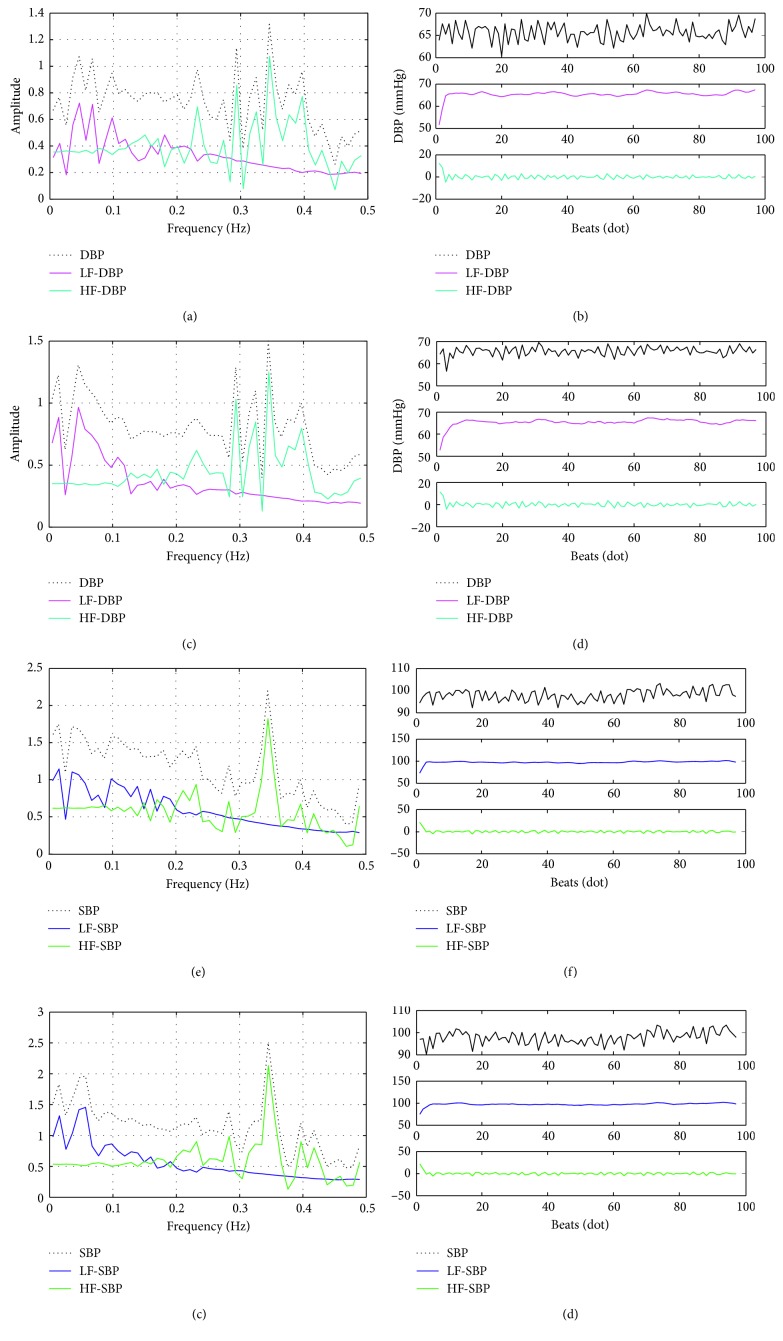
One exemplary reference BP signal decomposition results in time and frequency domain. (a) The reference DBP spectral decomposition. (b) The reference DBP. (c) The estimated DBP spectral decomposition. (d) The estimated DBP. (e) The reference SBP spectral decomposition. (f) The reference SBP. (g) The estimated SBP spectral decomposition and (h) the estimated SBP.

Figure 4 shows one segment for the time-frequency decomposition results of the reference and the corresponding estimated BP signal. It could be observed that the spectral decomposition results of the estimated BP are obviously different from those of reference BP, especially in the LF regions. Similarly, in the time domain, the differences between the estimated BP and the reference BP are more obvious in the LF region than those in the HF region. These results are consistent with the research results of Ma and Zhang [26] and Liu et al. [27], i.e., the worse performance of PTT in LF-BP estimation.

Then, the estimation errors of the PTT-based SBP/DBP were calculated in LF, HF, and overall as follows:

(11) $$\begin{array}{c} e_{\text{LFSBP}i}^{n}=\text{LF}{\hat{\text{SBP}}}_{i}^{n}-{\text{LFSBP}}_{i}^{n},\\ e_{\text{LFDBP}i}^{n}=\text{LF}{\hat{\text{DBP}}}_{i}^{n}-{\text{LFDBP}}_{i}^{n},\\ e_{\text{HFSBP}i}^{n}=\text{HF}{\hat{\text{SBP}}}_{i}^{n}-{\text{HFSBP}}_{i}^{n},\\ e_{\text{HFDBP}i}^{n}=\text{HF}{\hat{\text{DBP}}}_{i}^{n}-{\text{HFDBP}}_{i}^{n},\\ e_{\text{SBP}i}^{n}={\hat{\text{SBP}}}_{i}^{n}-{\text{SBP}}_{i}^{n},\\ e_{\text{DBP}i}^{n}={\hat{\text{DBP}}}_{i}^{n}-{\text{DBP}}_{i}^{n}, \end{array}$$

where *e* is the estimation error, $\hat{\left[\cdot \right]}$ is the estimation value, the rightmost subtrahend in the equations is the corresponding reference BP value, *n* represents the *n*th subject, and *i* is the *i*th data point.

**Figure 5:**
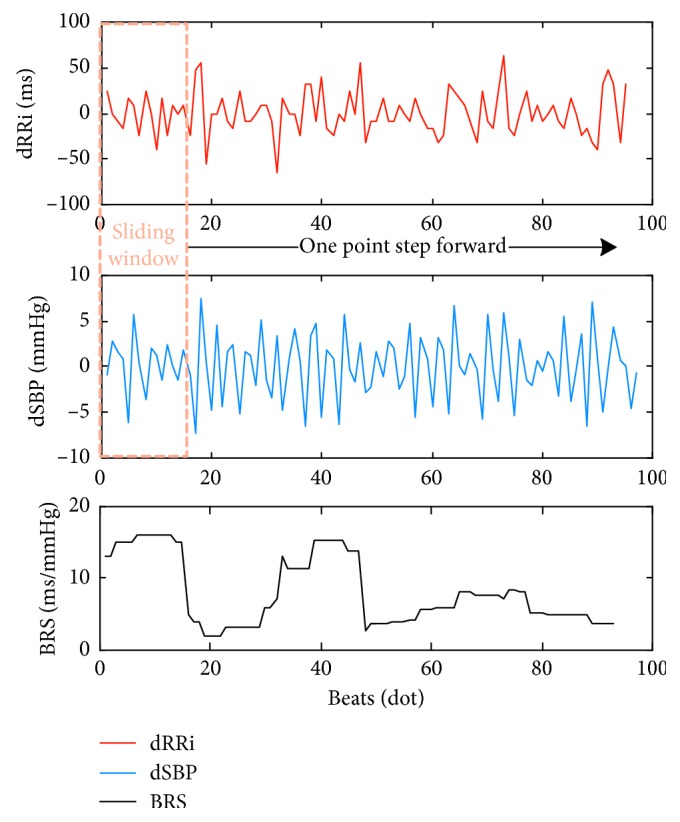
Dynamic BRS calculation. dRRi and dSBP are the first-order forward difference of RRi and reference SBP, respectively.

The ABR sensitivity is quantified by the clinical parameter baroreflex sensitivity (BRS) which is defined as the RRi variations as a reaction to the change of BP. The most widely applied BRS measuring method is the time-domain approach, in which BRS (measured in ms/mmHg) is calculated as the averaging regression coefficient between the first-order difference of RRi (measured in ms) and SBP (measured in mmHg) changes in the same direction (dRRi > 0, dSBP > 0 or dRRi < 0, dSBP < 0, i.e., 1st and 3rd quadrants) during a sliding window [35]. In this study, the BRS is dynamically calculated with a one-point-overlapping sliding window in size of 16 data points [35].  Figure 5 shows the process of dynamic BRS calculation. The details of the algorithm were described as the pseudocode in Table 2.

### 3.3. Correlation Evaluation

To confirm and pinpoint the influence of ABR sensitivity on the PTT-based BP estimation, the Pearson's correlation coefficients *r* (represented in terms of *μ* and SD) was utilized to calculate the correlation between the estimation error of PTT-based BP and BRS for each subject. Also, we applied the minimum absolute value (MAV) to test the significance of the correlation coefficients. The specific formulas of these criteria are given as follows:

(12) $$\begin{array}{c} r_{n}=\frac{\sum e\cdot \text{BRS}-\left(\sum e\sum \text{BRS}/M\right)}{\sqrt{\left(\sum e^{2}-\left({\left(\sum e\right)}^{2}/M\right)\right)\sum {\text{BRS}}^{2}-\left({\left(\sum \text{BRS}\right)}^{2}/M\right)}},\\ \mu =\frac{1}{N}\left(\overset{N}{\underset{n=1}{\sum }}r_{n}\right),\\ \text{SD}=\sqrt{\frac{1}{N}\overset{N}{\underset{i=1}{\sum }}{\left(r_{n}-\mu \right)}^{2}},\\ \text{MAV}=\min\left[\left|r_{n}\right|\right], \end{array}$$

where *n* is the *n*th subject, *N* is the number of subjects, *M* is the number of data points, and min[|·|] is the minimum absolute value.

The obtained data were contrastively analyzed stage by stage. At first, the overall correlation coefficients were compared between two groups: (1) *e*_SBP_ versus BRS and (2) *e*_DBP_ versus BRS. It is to confirm if there is an overall correlation between BRS and the estimation error of PTT-based BP. Then, the correlations are analyzed in LF and HF sections comparatively: (1) *e*_LFSBP_ versus BRS, (2) *e*_LFDBP_ versus BRS, (3) *e*_HFSBP_ versus BRS, and (4) *e*_HFDBP_ versus BRS. It is designed to further test if there is relativity between the ABR sensitivity and the failure of PTT in LF-BP estimation. In each stage, the significance test of the difference between two groups was implemented with a *T*-test. It was expected to verify and confirm the influence of ABR on PTT-based BP estimation with these contrastive analyses.

## 4. Experiment Results and Discussion

### 4.1. Overall Correlation Analysis

**Figure 6:**
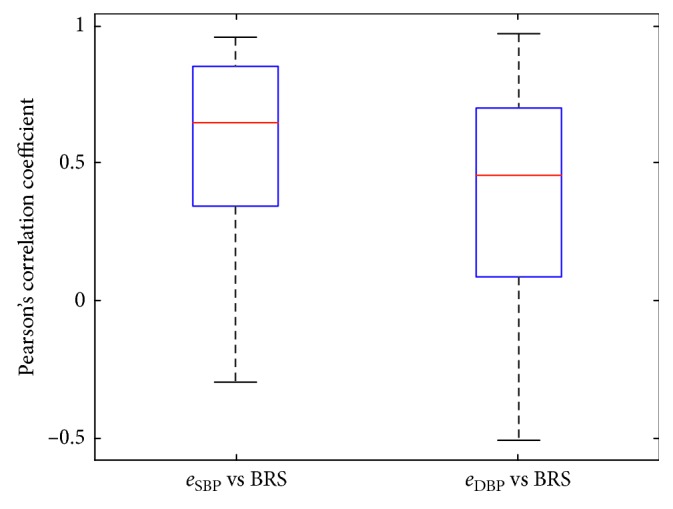
Boxplot of the overall correlation coefficients between the PTT-based SBP and DBP estimated error and BRS.

Figure 6 shows the boxplot of the overall correlation coefficients for two groups: (1) *e*_SBP_ versus BRS and (2) *e*_DBP_ versus BRS. The red line represents the median value. The bottom and top of the blue box are the first and third quartiles of data distributions, respectively. And, the black line represents 1.5 times the interquartile range of upper and lower quartiles. It is observed that the mean value of overall correlation coefficients in the group *e*_SBP_ versus BRS is higher than that in the group *e*_DBP_ versus BRS, indicating a closer relationship between *e*_SBP_ and BRS. However, it is worth noting that the correlation coefficients of each group are changing in both positive and negative regions. It reveals that the overall correlation for *e*_SBP_-BRS and *e*_DBP_-BRS is uncertain.

Table 3 lists the evaluation criteria for the correlation coefficients in detail. Especially, the MAV of the correlation coefficients in the group of *e*_SBP_ versus BRS and *e*_DBP_ versus BRS are 0.031 and 0.043, respectively. This near-zero value of MAV indicates the lack of correlation between ABR sensitivity and the overall PTT-based BP estimation error. It indicated that the ABR mechanism could not fully explain the estimation errors of PTT-based BP.

### 4.2. Correlation Analysis of the ABR Sensitivity and the Estimation Error for PTT-Based LF- and HF-BP

**Figure 7:**
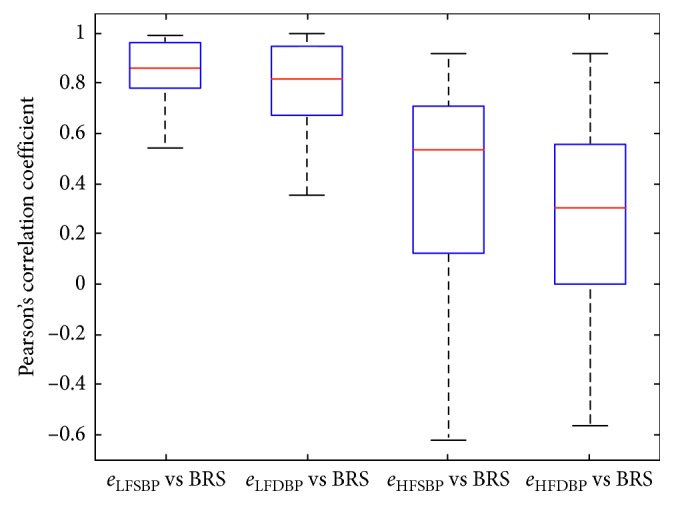
Boxplots of the correlation coefficients in LF (*e*_LFSBP_ versus BRS, *e*_LFDBP_ versus BRS) and HF (*e*_HFSBP_ versus BRS, *e*_HFDBP_ versus BRS) sections.

In addition, we compared the correlation coefficients between the PTT-based LF- and HF-BP estimation error and BRS, separately. As shown in Figure 7, it is observed that the correlation coefficients in the groups of *e*_LFSBP_ versus BRS and *e*_LFDBP_ versus BRS are more central around their mean values than those in the groups of *e*_HFSBP_ versus BRS and *e*_HFDBP_ versus BRS. Also, the mean values of the correlation coefficients in LF sections (*e*_LFSBP_ versus BRS, *e*_LFDBP_ versus BRS) are apparently higher than those in HF sections (*e*_HFSBP_ versus BRS, *e*_HFDBP_ versus BRS). In other words, there is a higher correlation between the ABR sensitivity and PTT-based LF-BP estimation error.

The detailed evaluation criteria for the correlation coefficients listed in Table 4 also indicate the higher correlation between the ABR sensitivity and PTT-based LF-BP estimation error. The mean value ± standard variation of the correlation coefficients in the group of *e*_LFSBP_ versus BRS and *e*_LFDBP_ versus BRS is 0.817 ± 0.038 and 0.411 ± 0.145, respectively. In contrast, the correlation coefficients for the group of *e*_HFSBP_ versus BRS and *e*_HFDBP_ versus BRS are only 0.403 ± 0.145 and 0.274 ± 0.154, respectively. The MAV value is 0.522 and 0.355 in the LF group of *e*_LFSBP_ versus BRS and *e*_LFDBP_ versus BRS, respectively. However, the MAV is only 0.071 and 0.080 in the HF group of *e*_HFSBP_ versus BRS and *e*_HFDBP_ versus BRS, respectively. In the *t*-test of the difference between the LF and HF class, significant differences were found (*p* < 0.01).

These results clearly suggested that there is a high correlation between the PTT-based LF-BP estimation error and ABR sensitivity. Nevertheless, no correlation has been found between the PTT-based HF-BP estimation error and ABR sensitivity. It is consistent with the results of qualitative analyses in Section 2.4.

## 5. Conclusion and Further Works

In this paper, we analyzed the poor performance of PTT in LF-BP estimation for the subjects before and after exercise. At first, based on the ABR model, the influence of ABR sensitivity on PTT-based BP estimation was analyzed. It was found that HR may have the unexpected effects on PTT in the LF section under the ABR regulation, which could help to explain the cause behind the failure of PTT in LF-BP estimation. Then, the physiological data from 42 volunteers were collected to verify this hypothesis through correlation analysis between the ABR sensitivity and the estimation error of PTT-based BP in different frequencies. For the overall correlation analysis (Table 3), no obvious correlation was found between the ABR sensitivity and the PTT-based SBP and DBP estimated error. The *r* value is 0.533 ± 0.109 and 0.411 ± 0.145 for the SBP and DBP group, respectively. However, in the comparative correlation analysis (Table 4), remarkable differences (*p* < 0.01) on the correlations of ABR sensitivity and PTT-based BP estimation error have been observed among LF and HF groups. Especially, in the correlation analysis of ABR sensitivity and the PTT-based LF-BP estimation error, very high *r* values of 0.817 ± 0.038 and 0.757 ± 0.069 are observed in the SBP and DBP group, respectively. In contrast, for the correlation of ABR sensitivity and PTT-based HF-BP estimation error, only *r* values of 0.403 ± 0.145 and 0.274 ± 0.154 are viewed in the SBP and DBP group, respectively. Moreover, it is worth noting that the MAV values in the LF group are all higher than those in the HF group.

These experiment results indicated that there is a definitely high correlation between the error of PTT-based LF-BP estimation and the ABR sensitivity for the subjects before and after exercise. To the best of our knowledge, this phenomenon has not been mentioned in the previous studies. This is also the most important finding in this study. It suggested that there is an ABR-related complex LF autonomic regulation mechanism on BP, PTT, and HR, which influences the estimation accuracy of PTT in LF-BP for the subjects before and after exercise. This finding also gave some possible inspirations to help constructing a better PTT-based BP estimation model that: (1) to weaken the influence of ABR, HR is an important parameter that should be considered in improving the accuracy of PTT-based LF-BP estimation; and (2) a weighted frequency-dependent model is required to better estimate BP from PTT. However, it should be noted that there are some limitations in this study. Due to the complexity of cardiovascular system, ABR could not be the only reason for the failure of PTT in LF-BP estimation. The details should be investigated with more different cardiovascular parameters from the subjects under different conditions in further work.

## Figures and Tables

**Table 1:** The experiment procedure.

Physiological conditions	Trial no.	Time (min)	Recording Length (min)
Acclimatization	1	10	0
Sitting rest	2	10	10
Treadmill running (8 km/h)	3	5	0
Sitting recovery	4	10	10

**Table 2:** The pseudocode of BRS calculation.

**Initialize**
The sliding-window start data-point number *w*=0
Searching the sliding window:
Set inside-window start data-point number *i*=0
** Repeat**
** ** *i*=*i*+1,
** if** (dRRi(*w*+*i*) < 0 **and** dSBP(*w*+*i*) < 0)
** **Register *i* in the **first-quadrant** array
** else if** (dRRi(*w*+*i*) > 0 **and** dSBP(*w*+*i*) > 0)
** **Register *i* in the **third-quadrant** array
** Until** *i*=16
Calculate the regression coefficient between dRRi (i) and dSBP (i) in the **first-quadrant** and **third-quadrant** separately: **R**_**1**_ and **R**_**2**_
** **BRS in the *w*th sliding-window is calculated as:
** **BRS *w* = (**R**_**1**_ + **R**_**2**_)/2, *w*=*w*+1.
**go to** Searching the sliding window;
until the last data-point is reached

**Table 3:** The overall correlation for BRS and BP estimation error.

Groups	Pearson's correlation coefficients (*r*) (*p* < 0.05)		
	*μ*	SD	MAV
*e*_SBP_ vs BRS	0.533	0.109	0.031
*e*_DBP_ vs BRS	0.411	0.145	0.043

**Table 4:** The correlation for BRS and BP estimation error in LF and HF sections.

Classes		Groups	Pearson's correlation coefficients (*r*)		
			*μ*	SD	MAV
*p* < 0.01	LF	*e*_LFSBP_ vs BRS	0.817	0.038	0.522
		*e*_LFDBP_ vs BRS	0.757	0.069	0.355
	HF	*e*_HFSBP_ vs BRS	0.403	0.145	0.071
		*e*_HFDBP_ vs BRS	0.274	0.154	0.080

## Data Availability

The physiological data used to support the findings of this study are available from the corresponding author upon request.
